# Dance on the Brain: Enhancing Intra- and Inter-Brain Synchrony

**DOI:** 10.3389/fnhum.2020.584312

**Published:** 2021-01-07

**Authors:** Julia C. Basso, Medha K. Satyal, Rachel Rugh

**Affiliations:** ^1^Department of Human Nutrition, Foods, and Exercise, Virginia Tech, Blacksburg, VA, United States; ^2^Center for Transformative Research on Health Behaviors, Fralin Biomedical Research Institute, Virginia Tech, Blacksburg, VA, United States; ^3^School of Neuroscience, Virginia Tech, Blacksburg, VA, United States; ^4^Graduate Program in Translational Biology, Medicine, and Health, Virginia Tech, Blacksburg, VA, United States; ^5^Center for Communicating Science, Virginia Tech, Blacksburg, VA, United States; ^6^School of Performing Arts, Virginia Tech, Blacksburg, VA, United States

**Keywords:** interpersonal coordination, improvisation, neurobehavior, shared intentionality, hyperscanning, neural coupling, neural synchrony, interpersonal brain synchronization

## Abstract

Dance has traditionally been viewed from a Eurocentric perspective as a mode of self-expression that involves the human body moving through space, performed for the purposes of art, and viewed by an audience. In this Hypothesis and Theory article, we synthesize findings from anthropology, sociology, psychology, dance pedagogy, and neuroscience to propose The Synchronicity Hypothesis of Dance, which states that humans dance to enhance both intra- and inter-brain synchrony. We outline a neurocentric definition of dance, which suggests that dance involves neurobehavioral processes in seven distinct areas including sensory, motor, cognitive, social, emotional, rhythmic, and creative. We explore The Synchronicity Hypothesis of Dance through several avenues. First, we examine evolutionary theories of dance, which suggest that dance drives interpersonal coordination. Second, we examine fundamental movement patterns, which emerge throughout development and are omnipresent across cultures of the world. Third, we examine how each of the seven neurobehaviors increases intra- and inter-brain synchrony. Fourth, we examine the neuroimaging literature on dance to identify the brain regions most involved in and affected by dance. The findings presented here support our hypothesis that we engage in dance for the purpose of intrinsic reward, which as a result of dance-induced increases in neural synchrony, leads to enhanced interpersonal coordination. This hypothesis suggests that dance may be helpful to repattern oscillatory activity, leading to clinical improvements in autism spectrum disorder and other disorders with oscillatory activity impairments. Finally, we offer suggestions for future directions and discuss the idea that our consciousness can be redefined not just as an individual process but as a shared experience that we can positively influence by dancing together.

## Introduction: Dance to Enhance Neural Synchrony

Rhythmic patterns are omnipresent throughout nature, such as the rising and setting of the sun, the movement of ocean waves, the beating of the heart, or the inhalation and exhalation of respiration (Strogatz, 2004). Natural systems are periodic, persistent, and often represented as cyclical waves (i.e., sine waves; Winfree, 1967). Along with this rhythmic nature, many natural systems are complex (Strogatz, 2004; Ma’ayan, 2017). Complex systems are open systems, adhere to non-linear dynamics, and are self-organizing in nature, moving from disorganization to organization (Kauffman, 1993; Corning, 1995; Kaplan and Glass, 1997). Examples of complex systems include the organization of DNA, insect colonies, or schools of fish in the sea (Ma’ayan, 2017). These systems are emergent, with the end outcomes often being unpredictable due to the non-linear dynamics that are at play (Kaplan and Glass, 1997). The brain is also a complex, self-organizing system, which organizes in activity patterns known as oscillations [e.g., theta wave (4–8 Hz); alpha wave (8–12 Hz); beta wave (12–30 Hz); Buzsáki, 2006]. This continuous rhythmic activity meaningfully encodes information and creates our conscious experience (Buzsáki, 2006; Gallotto et al., 2017; Cebolla and Cheron, 2019).

Recent theoretical work suggests that movement is inherent to or perhaps drives consciousness (Cebolla and Cheron, 2019). Rhythms of the body are important throughout life. The first action of the nervous system is movement, and it is through the spontaneous movement of the fetus that the body and brain can correctly develop. Early in life, motor movement actually propels cortical brain activity (called spindle oscillations; Khazipov et al., 2004; Buzsáki, 2006), and these movements help in the development of cognitive skills like language, as well as social and emotional intelligence (Zentner and Eerola, 2010; Cirelli et al., 2014; Trehub and Cirelli, 2018). Later, movement propels hippocampal and cortical oscillations, which increase synaptic plasticity, facilitate enhanced communication between brain areas, and optimize brain functioning throughout adulthood and into old age (Sirota and Buzsáki, 2005; Headley and Paré, 2017). That is, brain-body connectivity is bidirectional: oscillatory rhythms in the brain drive movement and movement drives oscillatory rhythms.

Dance, as a multifaceted movement form, is truly an intrinsic human behavior that emerges as early as infancy. Babies move in sync with musical rhythms, with the synchronicity between the movement and sound related to the experience of pleasure (Zentner and Eerola, 2010; Fujii et al., 2014; Trehub and Cirelli, 2018). This synchronicity of movement to music can also be seen on any dance floor. When humans hear music, they are driven to move in tune or entrain to the beat, with this rhythmic entrainment leading to positive affective states (Phillips-Silver et al., 2010; Trost et al., 2017). In this manuscript, we synthesize findings from anthropology, sociology, psychology, dance pedagogy, and neuroscience to propose The Synchronicity Hypothesis of Dance, which states that humans dance to enhance both intra- and inter-brain synchrony. We explore this idea through several avenues. First, we examine evolutionary theories of dance, which suggest that dance drives interpersonal coordination. Second, we examine fundamental movement patterns, which emerge throughout development and are omnipresent across cultures of the world. Third, we examine how each of the seven neurobehaviors increases intra- and inter-brain synchrony. Fourth, we examine the neuroimaging literature on dance to identify the brain regions most involved in and affected by dance. The findings presented here support our hypothesis that humans dance for the purpose of intrinsic reward (Richard et al., 2013; Robinson et al., 2016), which as a result of dance-induced increases in neural synchrony, leads to enhanced interpersonal coordination.

**Figure 1:**
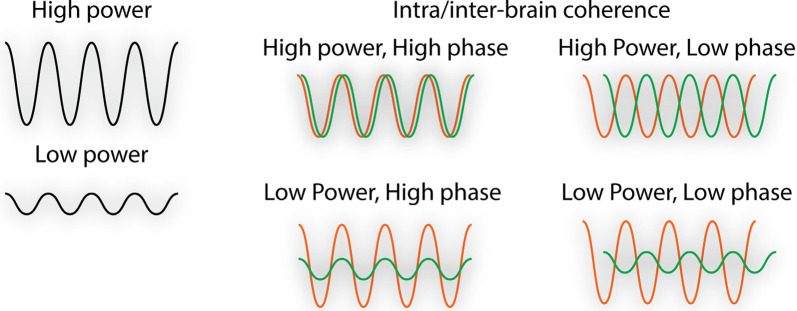
Illustration of the quantification of neural oscillations. Neural synchrony changes are reflected as: (1) increased power/amplitude; or (2) increased coherence or correlation between either the power and/or phase between multiple brain areas (intra-brain) or between people (inter-brain).

Throughout the manuscript, the term “neural synchrony” refers to oscillatory neural activity. Neural oscillations emerge as a result of population-level neuronal firing and enable effective communication within and between brain structures (Koepsell et al., 2010). Neuronal oscillations, recorded in humans primarily through the technique of electroencephalography (EEG), can be quantified in terms of power (i.e., amplitude) and coherence (i.e., correlated power and/or phase between multiple brain areas or between people; Figure 1). When we discuss intra-brain synchrony, we refer to coordinated neural activity or neural coupling within or between brain regions at an individual level. When we discuss inter-brain synchrony, we refer to the neural coupling between people. Inter-brain synchrony is measured by the hyperscanning technique, which is a term coined in 2002 that refers to the simultaneous recording of brain activity between two or more individuals. To explore intra- and inter-brain synchrony, we include studies that have recorded brain activity utilizing EEG, functional magnetic resonance imaging (fMRI), or functional near-infrared spectroscopy (fNIRS), which each utilize unique statistical methods to quantify neural coupling but use the general approach of correlating neural activity between brain regions or between people (Hasson et al., 2004; Liu et al., 2018a). Though EEG measures neural activity directly through population-level neuronal firing, fMRI and fNIRS measure neural activity indirectly through changes in blood-oxygenation level (i.e., the hemodynamic response). Each of these techniques has unique advantages, with EEG having an excellent temporal resolution, fMRI having an excellent spatial resolution, and fNIRS being superior to fMRI in terms of its temporal dynamics and tolerance for motion.

## The Synchronicity Hypothesis of Dance

Historically, dance as an art form has been viewed by Western scholars from a limited and Eurocentric perspective (primarily placing value on dances with Western European lineage and aesthetic preferences, such as ballet; Amin, 2016; Walker, 2019). However, when we think of dance more comprehensively and inclusively, we can include other contemporary movement forms such as hip hop, improvisation, and authentic movement, as well as non-Western traditional folk dances. To help us think about dance, we must expand our perspective of dance as a pure art form.

Arriving at one concrete definition of dance is difficult, as dancing serves an array of purposes for human populations in vastly different cultural contexts. Worldwide, dance has traditionally been integral to religious rituals and rites of passage (Hanna, 1988). This can be seen in contexts as disparate as the Salpuri shamanic dances of Korea, the Sun Dance of the American Plains Indians, and even in the ecstatic dances of the Dionysian cults of ancient Greece (Lawrence, 1993; Lonsdale, 2001; Park et al., 2002). Previous definitions of dance have focused on anthropological and sociological perspectives (Hanna et al., 1979; Reed, 1998; Kaeppler, 2000). For example, dance anthropologist, Joann Kealiinohomoku, defines dance as “a transient mode of expression performed in a given form and style by the human body moving in space.” She goes on to note that “dance occurs through purposefully selected and controlled rhythmic movements; the resulting phenomenon is recognized as dance both by the performer and the observing members of a given group” (Williams, 1976). Previous scientific work has proposed several neural and biobehavioral functions of dance including: (1) attention focus/flow; (2) basic emotional experiences; (3) imagery; (4) communication; (5) self-intimation; and (6) social cohesion (Christensen et al., 2017). Here, we create a new definition of dance that encompasses both of these lenses, and that focuses on dance as a human behavior that emerges from the brain: The Neurocentric Definition of Dance (Box 2). This definition provides a neuroscientific framework from which to investigate how the brain manifests dance and movement forms, as well as the effects of dance on the brain.

Box 1 **The neurocentric definition of dance.**

Dance encompasses an unlimited array of movement patterns that: (1) are spontaneously or intentionally generated; (2) are manifested for the purpose of ritual, performance, or social interactions; and (3) engage a diverse network of brain regions that support neurobehavioral processes in seven distinct areas:

• Sensory
• Motor
• Cognitive
• Social
• Emotional
• Rhythmic
• Creative.

Box 2 **Fundamental movement patterns codified in dance.**

Taking note of the developmental movement patterns (described above) and following from the work of Rudolf Laban and Laban Movement Analysis (LMA), Irmgard Bartenieff developed a set of movement principles known as the Bartenieff Fundamentals (Berardi, 2004). Bartenieff noted a series of six movement patterns that humans move through continually, first as infants then later in varying forms throughout the life cycle. These patterns were later codified by her student Peggy Hackney as the *Fundamental Patterns of Total Body Connectivity*. These six neurodevelopmental patterns of movement are described below and visually presented in Figure 3.

**Figure 2:**
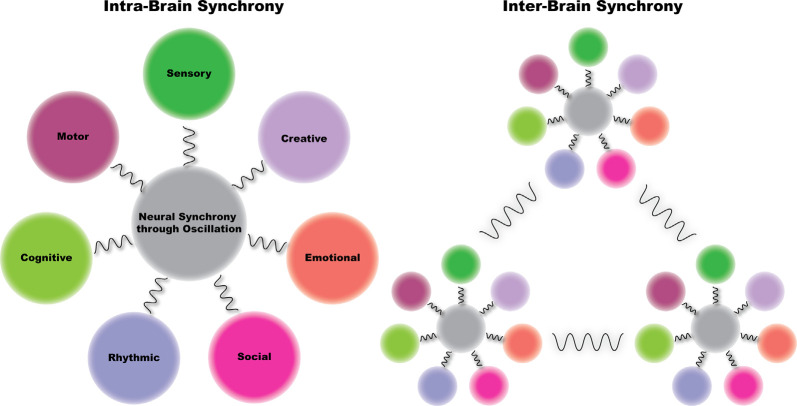
The synchronicity hypothesis of dance—we hypothesize that dance enhances neural synchrony in brain regions supporting seven neurobehavioral areas: sensory, motor, cognitive, social, emotional, rhythmic, and creative. Further, we hypothesize that when we engage in dance with others, brain dynamics between individuals become synchronized. That is, dance enhances both intra- and inter-brain synchrony. Finally, we posit that we engage in dance for the purpose of intrinsic reward, which as a result of dance-induced increases in neural synchrony, leads to enhanced interpersonal coordination.

**Figure 3:**
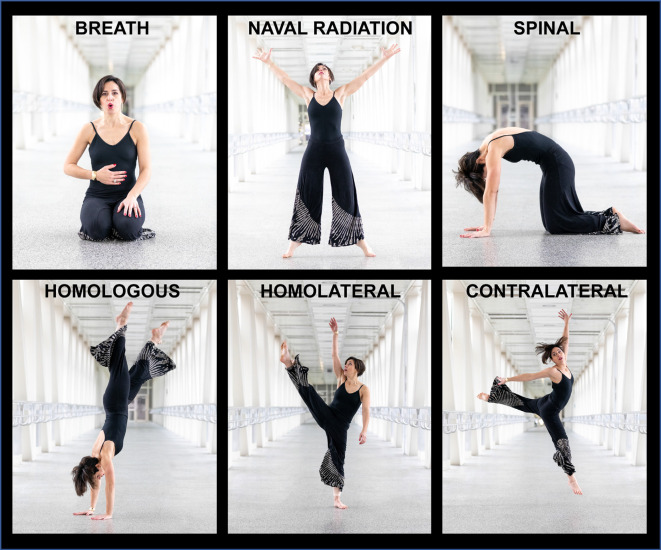
Fundamental patterns of total body connectivity: Bartenieff and Hackney’s six *Fundamental Patterns of Total Body Connectivity* are demonstrated: breath, naval radiation (core-distal), spinal (head-tail), homologous (upper-lower), homolateral (body half), and contralateral (diagonal). Photo credit: Kathryn Butler (https://www.kathrynbutlerphotography.com).

**1. Breath**: The most important adaptation after birth is the transition to breathing, which occurs approximately 10 s after birth (Hillman et al., 2012). Breath is the first of the movement patterns to develop in a newborn and is essential for life. Respiration occurs in the body on a micro-level *via* cellular respiration and at a macro-level *via* the functioning of the lungs. Recently, respiration has also been linked to global brain oscillations, concomitantly occurring with theta oscillations (Tort et al., 2018a,b). For dancers, breath is especially important. Respiration rate and the increased exchange of oxygen and carbon dioxide dictates the ability to engage in vigorous movement. Additionally, breath allows for the phrasing of movement as well as the growing and shrinking of the body in space, which helps shift the musculoskeletal structure of the body.

**2. Naval Radiation (Core-Distal)**: Naval radiation patterns of movement are based on the connectivity between the center (core) of the body, and the distal ends of the body (hands/feet/head/tail). During these movements, the core is engaged, and movement radiates outward from this point, with movements contracting in and radiating out. These patterns of movement are observed in starfish and other sea creatures that use radiation to locomote. In human development, these patterns of movement can be observed in human infants with the Moro reflex, which can be seen when a baby is startled (Futagi et al., 2012). In a dance context, core-distal connectivity includes any type of movement in which the core provides stability to facilitate mobility through the distal ends of the body (e.g., jumping jacks).

**3. Spinal (Head-Tail)**: Spinal patterns are based on the connectivity of the central axis, with the endpoints being the head and tail. This includes mobility of the vertebral column but also includes the movement of other axial structures, such as the digestive tract and the spinal cord. Spinal movements are associated with the horizontal plane and incorporate basic movements of the spine including flexion, extension, rotation, and lateral flexion. Head-tail connectivity is expressed as curvature in the spine and can be seen in cat-cow pose (*Chakravakasana*) in yoga. In jazz dance, a “body roll” or any undulating movement of the spine exhibits head-tail connectivity.

**4. Homologous (Upper-Lower)**: Homologous patterns are based on the differentiation between the upper and lower body. In these movement patterns, the upper body is engaged in dynamic movements, while the lower body provides stability and locomotion. These movements manifest in the sagittal plane and are often symmetrical such as jumps, push-ups, or burpees. The upper and lower body may move in opposition to provide a sense of balance, such as in balancing postures where the legs ground down while the arms reach up. Conversely, any “inversion” in which the weight is primarily held by the upper body engages upper-lower connectivity (e.g., handstands).

**5. Homolateral (Body Half)**: Homolateral patterns are based on the differentiation between the right and left sides of the body. In these movement patterns, one side of the body stabilizes while the other side of the body moves. These movements exist in the vertical plane and are associated with asymmetrical movements. In humans, this movement pattern emerges with the ability to crawl and can be seen in hopping or skipping. Later, this body half ability helps develop horizontal eye tracking, which is necessary for early literacy skills (Karatekin, 2007).

**6. Contralateral (Diagonal)**: Contralateral patterns are based on the connectivity of an upper limb to an opposite lower limb and involve the crossing of the center of the body. Diagonal patterns are the most evolutionary and developmentally advanced forms of movement and include complex human movements such as walking, spiraling, and turning. These diagonal forms help develop vertical eye tracking also necessary for early literacy skills.

Cross-lateral connectivity (i.e., contralateral/diagonal) is the culminating exercise in Bartenieff’s series of fundamental movement patterns. Bartenieff hypothesized that to fully prepare the brain and body for cross-lateral integration (i.e., the ability to cross the midline of the body), it is important to first also internalize head-tail, homologous, and homolateral movement patterns. By repeatedly returning to and refining these six patterns, Bartenieff and her students hypothesized that individuals could achieve more easeful, efficient, pain-free, and enjoyable movement. These patterns of movement are present in many forms of dance training and are included intentionally in some contemporary somatic practices such as Body-Mind Centering (developed by Bartenieff’s student Bonnie Bainbridge Cohen). At present, limited research has been conducted to investigate the effectiveness of these innate patterns of movement to brain function and physiology (Chatfield and Barr, 1994). The primary work in this field has focused on the connection between movement patterns, quantified through LMA, and their connection to emotional expression or the expressive quality of movement (Bernardet et al., 2019; Melzer et al., 2019; Tsachor and Shafir, 2019). More recently, LMA in combination with EEG has been used to extract neural signatures linked to expressive human movement (Cruz-Garza et al., 2014). Additionally, compared to traditional physical activity, movement programs that include Bartenieff Fundamentals have also proven more effective at improving cognitive issues in individuals with mild cognitive impairment (Kim, 2018). More research is needed to fully understand how engagement in these innate patterns of movement can improve motor and other neurobehavioral functions in both healthy and clinical populations.

This definition sets the stage for our neurocentric, evolutionary hypothesis of dance. As we have discussed previously, the brain spontaneously generates states of coordinated activity that lead to our experience of consciousness. Heightened coordinated brain states are associated with an enhanced ability to learn and remember information, heightened affective states, enhanced flow states, and higher levels of prosociality (Buzsáki, 2006), which we will discuss in more depth below.

We hypothesize that dance evolved as a spontaneous process to drive coherent electrical activity between brain regions. As the physical body becomes tuned to either external (e.g., music) or internal (e.g., breathing) rhythms, these rhythms entrain regions of the brain connected to the external world (auditory and sensory) and subsequently recruit other, more internally focused brain areas (motor, cognitive, and emotional). This entrainment creates enhanced synchronicity (i.e., increased power and coherence) between these areas, promoting enhanced neurobehavioral effects in sensory, motor, cognitive, social, emotional, rhythmic, and creative brain regions. We further hypothesize that when we engage in dance in a group, brain dynamics between individuals in the group become synchronized—that is, dance enhances both intra- and inter-brain synchrony. We term this The Synchronicity Hypothesis of Dance. We engage in dance for intrinsic reward to drive brain synchrony both within and between individuals, which leads to the behavioral outcome of enhanced interpersonal coordination (Figure 2).

## Evolutionary Purpose of Dance: Driving Interpersonal Coordination

One current evolutionary theory of dance posits that dance evolved as a form of interpersonal coordination, which includes both imitation and synchrony (Laland et al., 2016). Imitation or mimicry (used interchangeably in this text) refers to the matching of movement, whereas synchrony refers to the matching of time (Bernieri and Rosenthal, 1991; Hove and Risen, 2009; Chartrand and Lakin, 2013). Interpersonal coordination has been evolutionarily selected for its important role in social cohesion or bonding (Chartrand and Lakin, 2013); these behaviors help connect the self to others, and recent studies have shown that they drive neural coupling (Bernieri and Rosenthal, 1991; Hasson et al., 2012; Hasson and Frith, 2016). Other theories on the evolution of dance and other creative art forms exist but are beyond the scope of this review (Morriss-Kay, 2010; Sigaki et al., 2018; Zaidel, 2018, 2020; Savage, 2019; Harvey, 2020). In this section, we discuss the evolutionary advantages of interpersonal coordination, and by viewing dance through this lens, we see that dance is a complex physical activity that involves mimicry and synchrony in a variety of neurobehaviors.

Humans are exceptional imitators, though the ability to imitate is also seen in other species, such as songbirds and insects (Tchernichovski and Marcus, 2014; Duranton and Gaunet, 2016; Maria and Shizuka, 2018). As humans, some of our first successful interactions with the world depend on imitation. Imitation is an important feature of human development because it is through imitation that we develop social cognition, which enables us to understand another’s thoughts and feelings and share our conscious experiences with one another. During development, early social interactions between mothers and infants help shape later socioemotional functions (Prochazkova and Kret, 2017; Miller et al., 2019). For example, during language acquisition in infants, mothers often speak in exaggerated tones and facial expressions to emphasize the sounds and movements of novel words, a behavioral phenomenon known as motherese (Nelson et al., 1989; Falk, 2004). As we grow, imitation continues to be an important behavioral skill, as it enables us to connect and develop social bonds. As humans, we often imitate others’ speech, movements, gestures, facial expressions, and eye gaze (Duranton and Gaunet, 2016). Also, evidence is accumulating that human mimicry goes beyond vocal and motor mimicry to include synchrony of heart rate, pupil diameter, blushing, crying, and yawning (Kleinbub, 2017; Palumbo et al., 2017). This phenomenon is known as automatic or autonomic mimicry, and it is thought that through these subtle or unconscious interactions, we share one another’s emotional landscape, which is known as emotional contagion and helps to develop our sense of empathy (Hess and Fischer, 2013; Prochazkova and Kret, 2017).

Humans, like other social animals, live in groups, and the success (e.g., physical, emotional, reproductive, financial) of the group relies on dynamic social interactions between its members (Alexander, 1974; Rubenstein, 1978). The development of socioemotional processes is therefore evolutionarily advantageous because they allow us to predict another’s actions so we can adaptively respond to various group situations, including those that are welcoming or threatening. Also, these skills help us effectively integrate into other social environments, including those of family, friends, and work, which ultimately add to our successful survival. These ideas have been synthesized by Shamay-Tsoory et al. (2019) into a model of social alignment, whereby synchrony of movement, cognition, and emotion work together to drive our sense of social alignment or connectivity to a group. The experience of social alignment is in itself intrinsically rewarding, which drives us to engage in prosocial behaviors. Further, when we sense that we are socially misaligned, we will correct our actions to drive heightened social alignment.

We posit that dance may be an expansion of this process. Dance incorporates many aspects of interpersonal coordination, including touch, eye gaze, sensory-motor interactions, rhythmic or in tandem movement, physical movement coordination, facial expressions, or emotional qualities, and even synchronization with other physiological parameters, such as breathing, heartbeat, and sympathetic tone. To this point, research has demonstrated that compared to non-dancers, dancers have enhanced interpersonal coordination skills (Sofianidis et al., 2012; Washburn et al., 2014). Interpersonal coordination is a key skill for a dancer because to effectively pick up a choreographic sequence (and get chosen for a role), the dancer needs to be able to imitate movement patterns, as well as the cadence of the movement (either to coordinate it to rhythmic patterns of sound or an internally generated rhythm). Dancers are trained to do this by watching others move (e.g., choreographer, teacher, other dancers), by watching themselves in the mirror, and by using imagery to sense how the movement should be generated. Dancers move closer to generating the correct movement sequences by correcting their actions based on feedback from the teacher, based on the feedback of watching themselves in the mirror, and by mentally visualizing the correct patterns of movement. We hypothesize that dance training enhances a series of neurobehaviors, which in turn contributes to enhanced interpersonal coordination skills. Throughout this manuscript, we demonstrate how these skills are related to enhanced neural synchrony at both the individual and group levels.

## Fundamental Movement Patterns in Dance and Their Relationship to Neural Synchrony

Innate patterns of movement emerge throughout development, are omnipresent throughout cultures of the world, and have been codified in dance technique. Here, we describe how these innate movement patterns develop and how they relate to the emergence of neural synchrony.

### Dance Throughout Development

Throughout development, a series of fundamental movement patterns or stages of motor development emerge, which we will briefly describe here. During and following birth, breath is a key factor in healthy motor development. The adaptation to independent breath is an imperative and complex factor in the transition from intrauterine to extrauterine life (Hillman et al., 2012). After breathing, lying is one of the first active positions demonstrated by newborn babies (Teitelbaum et al., 1998). Following lying comes the stage of righting from the supine to prone position (Teitelbaum et al., 1998). This occurs around the age of 3 months, whereby the infant will roll over from the back to the stomach and involves rotation along the longitudinal axis of the body. In the earliest stages, the pelvis turns first followed by the trunk, shoulders, and head. By 6 months, this movement pattern can be reversed, beginning with the head and ending with the tail—a phenomenon known as cephalic dominance. Sitting comes next around the age of 6 months, where equilibrium can be maintained through equal distribution of weight on the two ischia and coccyx (i.e., the sitting bones; Teitelbaum et al., 1998). At this point, the infant can engage in additional movement in the head, trunk, upper limbs, and hands, while maintaining stability in the sitting position. During this time, infants begin to develop the ability to rhythmically entrain to music. They can move rhythmically to music, exhibiting tempo flexibility, or the ability to change their movement in tune with the beat (Zentner and Eerola, 2010). Research suggests that this early form of dancing helps develop our interactions with the external world, including social and emotional development (Zentner and Eerola, 2010; Cirelli et al., 2014; Trehub and Cirelli, 2018). Crawling is the next stage, which may begin around the same time as sitting. When crawling is achieved, the infant moves forward on the hands and knees, with equal distribution on all four limbs, and the arms and thighs move parallel to the midline of the body (Teitelbaum et al., 1998). Standing develops around 8–10 months, where the infant can rise and stand for a few minutes, often supporting themselves against objects in the environment. Finally, walking emerges through three distinct stages governed by the use of the legs in a proximal to distal fashion (i.e., from the thighs to the feet; Teitelbaum et al., 1998). During waddling, the thigh is the only portion of the leg that actively moves, and the lower leg and foot are carried passively. Eventually, the child has full use of the feet and can take steps efficiently by equally shifting weight back and forth between the left and right sides of the body. Mastery of these movement patterns continues to develop through adolescence and into adulthood and is linked to and dependent upon coordinated brain activity.

During these critical periods of development, synchronized neural oscillations in both low (delta, theta, and alpha) and high (beta and gamma) frequency bands are necessary for the coordinated activity to emerge at both the brain and behavioral levels (Uhlhaas and Singer, 2010). Specifically, during neonatal development, involuntary movements of the fetus drive a pattern of cortical activity characterized by alpha-beta oscillations nested within a delta wave (Khazipov et al., 2004; Milh et al., 2007; An et al., 2014; Whitehead et al., 2018). These neural synchrony patterns help coordinate activity between sensory and motor regions, providing proper somatotopic mapping and sensorimotor processes (Tiriac et al., 2014). Throughout development, oscillations shift from lower to higher frequencies (i.e., increased gamma power) and develop heightened synchronicity (i.e., coherence or phase-locking value), with this process continuing into adulthood (Takano and Ogawa, 1998; Benasich et al., 2008; James et al., 2008; Müller et al., 2009; Poulsen et al., 2009; Uhlhaas et al., 2009; Cho et al., 2015; Marek et al., 2018). Recent research has shown that oscillatory activity in both somatosensory and motor cortices change throughout aging (e.g., increased post-movement beta rebound) and may underlie improvements in somatosensory processing and motor learning and performance (Gaetz et al., 2010; Espenhahn et al., 2019; Gehringer et al., 2019).

### Dance Across Cultures

**Figure 4:**
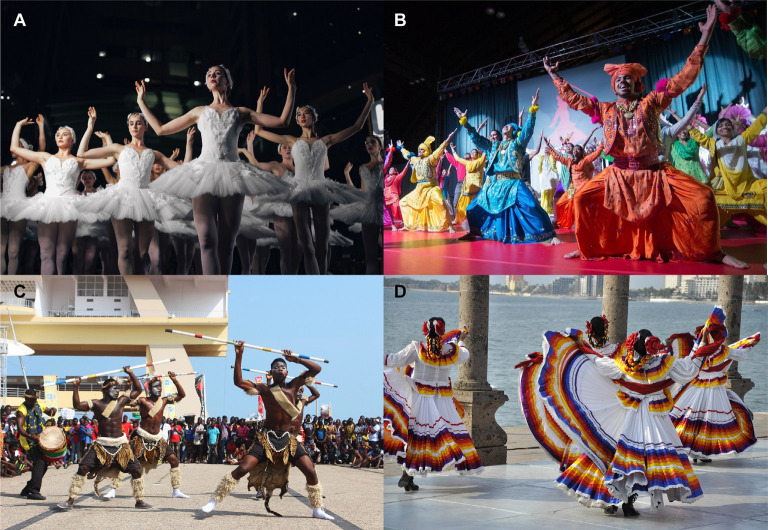
Fundamental patterns of movement are evident in dance forms throughout the world. This series of images demonstrates how naval radiation (core-distal) movements are present throughout various dance forms, including **(A)** ballet, a European dance form; **(B)** bhangra, an Indian dance form; **(C)** traditional Zulu dance, an African dance form; and **(D)** folkloric dance, a Mexican dance form. Photo credits: **(A)** Michael Afonso *via* Unsplash; **(B)** Itsachen *via* Creative Commons; **(C)** Fquasie *via* Creative Commons; **(D)** Lisette Lebaililif *via* Creative Commons.

Often termed a universal language, dance can be found in all cultures throughout the world. Though each culture has its expressive forms of dance, innate, or fundamental patterns of movement can be seen throughout these dance forms (Figure 4). Diversity in dance forms is created by differences in style and execution while using the same movement patterns (Alves, 2013). In addition to being a predictable facet of human development in the first 3 years of life, the movement patterns codified by Bartenieff and Hackney can be observed in a variety of dance forms across human cultures. For example, these patterns are present in the explosive naval radiation and homologous movements in West African traditional dance forms as well as the subtle homolateral and contralateral movements in the folk dances of Eastern Europe.

By taking a deeper look at one specific type of dance, we can see how these fundamental movement patterns are woven inextricably throughout the form. In Argentine tango, for example, the use of breath is an integral mechanism in partner cueing and phrasing. Core-distal connectivity is imperative, as the dancers radiate and reach away with grand arm and leg gestures, then return to the safety of their partner unit. Head-tail connectivity is apparent as dancers arch their backs, extending the spine dramatically into a low dip. To seamlessly lead and follow, effective upper-lower connectivity is key; dancers push and pull against each other with the upper body and yield their weight into the floor with the lower body. When transferring into an open embrace, dancers must utilize one body half independently from the other. Finally, to master the complicated footwork required of Argentine tango, effective use of diagonal movement patterns must be mastered.

## The Neurobehavioral Functions of Dance and Their Connection to Neural Synchrony

Along with physical virtuosity, including strength, flexibility, balance, limb coordination, and gross and fine motor control, dance is a performance art and requires a skill set that includes aesthetic, affective, communicative, and social elements (Yarrow et al., 2009). Dancers must learn and execute complex movement sequences through either verbal direction from a choreographer or visual observation from other dancers. Dancers must also execute movements in a particular time sequence, often in response to musical or rhythmic cues. In an ensemble, dancers must also remain aware of the movements of other dancers as they move in synchrony or execute specifically timed movements in response to other dancers. Therefore, effective dancing requires high levels of functioning across several different cognitive domains. Indeed, recent research has shown that compared to non-dancers, dancers display enhanced cognitive abilities, as well as distinct structural and functional brain changes that support these abilities (Bläsing et al., 2012; Burzynska et al., 2017). This section highlights how dance engages each neurobehavior highlighted in Box 1 and describes how growth and development in this neurobehavioral area relate to neural synchrony changes.

### Sensory

Touch, especially gentle touch, is important for social and emotional development, helps ease discomfort and pain, alleviates stress, is experienced as pleasurable, and increases positive affective states (Hertenstein et al., 2006; Korosi and Baram, 2010; Perini et al., 2015; Suvilehto et al., 2015; Krahé et al., 2016; Liljencrantz et al., 2017; Pawling et al., 2017; Goldstein et al., 2018). Additionally, studies using touch therapy, such as massage, have shown that touch is effective in relieving clinical symptoms in preterm infants and adults with pain disorders, such as rheumatoid arthritis and fibromyalgia (Field et al., 2010; Field, 2014; Hathaway et al., 2015). Dance training often involves the intentional activation of the somatosensory system, which is activated through stimulation of our sensory receptors, including mechanoreceptors in the skin (e.g., Meissner’s corpuscles, Pacinian corpuscles, and Ruffini endings), and proprioceptors in the muscle (e.g., muscle spindles and Golgi tendon organs). For example, a dance class may involve exercises where the whole body is placed on the floor and the dancer attempts to stimulate all parts of the skin, paying deep attention to the sensory experience. This practice is often accomplished with the eyes closed, to bring focus to the internal experience, and is intended to enhance the felt presence of the body in space. Alternatively, a dancer may engage in tactile stimulation of another dancer’s body by providing gentle massage with the hands or sensory exploration utilizing a range of body parts (e.g., feet, knees, elbows, and back). Possibly as a result of this sensory perception training, dancers show increased proprioceptive abilities (Kiefer et al., 2013; Volkerding and Ketcham, 2013).

Stimulation of the somatosensory system, as well as other sensory systems, is linked to enhanced neural oscillatory activity (Koepsell et al., 2010). For example, in rodents, the act of whisking or sensing the environment with the whiskers enhances neural synchrony, with the timing of the phase encoding spatial information about the environment (Ahissar et al., 2000; Szwed et al., 2003; Brecht, 2006). Additionally, non-human primate studies using single-unit recordings have shown that during intentional attending to tactile stimuli, as is practiced in dance, neural synchrony increases in sensory cortical areas, such as the anterior and lateral parietal cortices (Murthy and Fetz, 1992; Steinmetz et al., 2000; Roy et al., 2007; Delhaye et al., 2018). Somatic attention tasks in humans have also been shown to increase the intra-brain coupling in the gamma band (35–45 Hz) between contralateral prefrontal and parietal regions (Desmedt and Tomberg, 1994). Interesting new work in human romantic partners used a hyperscanning EEG approach to examine the effect of social touch (i.e., hand holding) on the experience of pain, with one partner experiencing the pain (target) and one partner watching (empathizer). Social touch increased inter-brain synchrony, specifically in the alpha band (8–12 Hz), with this effect being significantly related to both touch-related analgesia and the partner’s empathic accuracy (Goldstein et al., 2018). This study was followed up using a joint fMRI approach, finding that when the target experienced the pain, the empathizer showed similar activation in the inferior parietal lobe, an area of the action observation network (Korisky et al., 2020). Recent research indicates that enhanced neural synchrony in one sensory system has a cross-modal influence on other sensory systems (e.g., somatosensory to visual), suggesting that these oscillations may play a role in integrating our sensory experiences (Cooke et al., 2019; Bauer et al., 2020).

### Motor

As discussed earlier, motor movement is necessary for the correct development of cortical networks and for consciousness to emerge (Cebolla and Cheron, 2019). Both rodent and human studies have shown that motor movement in the form of physical activity significantly improves affective state and cognitive functioning and is beneficial for a range of neurodegenerative and neuropsychiatric disorders (Basso and Suzuki, 2017; Vivar and van Praag, 2017; Voss et al., 2019; Liu et al., 2019). Of all the neurobehaviors examined, dance most clearly activates the motor system, with technique training incorporating skills to enhance both gross and fine motor movements. Dance training early in life has been shown to enhance motor development and improve balance, equilibrium, postural control and alignment, range of motion, fine motor skills, and the planning and sequencing of movement, known as praxis (Golomer et al., 1999; Rein et al., 2011; Bläsing et al., 2012; Sirois-Leclerc et al., 2017; de Vasconcellos Corrêa Dos Anjos and Ferraro, 2018). Also, dancers optimize motor synergies (i.e., efficiently combine the movements in related joints; Latash, 2010), which lead to reduced muscular tension and increased accuracy when executing movements (Thullier and Moufti, 2004; Lepelley et al., 2006).

Examinations of the brain during or after physical activity lend support to the idea that motor movement enhances intra-brain synchrony. Specifically, enhanced cortical synchrony in different frequency bands (e.g., delta, theta, alpha, and beta) has been linked to the preparation, execution, perception, and imagining of movement (Zarka et al., 2014; Cevallos et al., 2015; Ewen et al., 2015; Tomassini et al., 2015). Most prominently, work in rodents has shown that during spatial navigation, wheel, and treadmill running, hippocampal theta increases (Buzsáki and Moser, 2013). Because the prefrontal cortex receives neuronal input and therefore activity from the hippocampus (Preston and Eichenbaum, 2013; Li et al., 2015b), prefrontal cortical neurons exhibit similar predictive or preparatory behavior during locomotion (Fujisawa et al., 2008). Similar to findings in rodents, human hippocampal theta activity occurs during movement and is positively correlated with movement speed (Aghajan et al., 2017; Bohbot et al., 2017; Yassa, 2018). Additionally, acute and chronic exercise in humans leads to increased oscillatory activity across a range of frequencies (measured with EEG) and increased functional connectivity (measured with fMRI) in brain regions associated with affect and reward processing, learning and memory, and attention and executive function (Crabbe and Dishman, 2004; Voss et al., 2010, 2016; Weng et al., 2017; Li et al., 2017).

Of relevance, synchronization of movement between individuals has been shown to enhance mood, memory performance, coordination, cooperation, affiliation, and altruistic behavior (Macrae et al., 2008; Hove and Risen, 2009; Wiltermuth and Heath, 2009; Valdesolo et al., 2010; Valdesolo and Desteno, 2011; Tschacher et al., 2014). For example, one study examined the effect of physical synchronization on inter-brain synchrony using fNIRS. Participants engaged in either synchronous or asynchronous movement of the right arm and then engaged in a teaching-learning task. The synchronous movement led to enhanced inter-brain synchrony in the lateral prefrontal cortex, with this effect being positively correlated to the level of rapport between the two participants (Nozawa et al., 2019). Technological advancements in group-dynamic brain recordings will be needed to study inter-brain synchronization between two or more simultaneously moving humans, such as occurs in dance.

### Cognitive

Cognitive processes develop in close conjunction with motor processes and are supported by coherent neural activity (Diamond, 2000; Fries, 2005). Dance is a complex form of physical activity in that it incorporates the cognitive processes of learning and remembering choreographic sequences. Dance and choreography have been linked to a range of cognitive functions, including attention, imagery, problem-solving, short- and long-term memory, and declarative and procedural memory (Stevens et al., 2003; Stevens and McKechnie, 2005; Sevdalis and Keller, 2011; Carey et al., 2019; Stevens et al., 2019). For example, dancers show excellent recall for complex motor movement sequences (Stevens et al., 2019), a skill that is inherent to dance training and reflects improved learning and memory performance. Additionally, compared to non-dancers, dancers demonstrate an enhanced ability to mentally rotate images (Bonny et al., 2017), a task that depends on spatial processing abilities. Finally, recent work in elderly adults has found that dance enhances prefrontal cortex-dependent executive functioning in areas including planning, working memory, and cognitive flexibility (Kosmat and Vranic, 2017; Noguera et al., 2020).

Cognitive processing has been linked to neural synchrony, especially in the theta frequency range. Specifically, theta oscillations are thought to facilitate memory formation (Buzsáki and Moser, 2013). Theta activity aids in the cognitive processing of sequential experience [including spatial (Skaggs and McNaughton, 1996), auditory (Aronov et al., 2017), and temporal aspects (MacDonald et al., 2011)] by organizing sequences of neuronal activity (Dragoi and Buzsáki, 2006), which are then repeated during subsequent sleep periods (Wilson and McNaughton, 1994; Siapas and Wilson, 1998; Lee and Wilson, 2002). In humans, the encoding of memories is positively correlated with theta amplitude (Lega et al., 2012), and theta induction through transcranial slow oscillation stimulation or transcranial magnetic stimulation supports memory encoding (Kirov et al., 2009; Tambini et al., 2018). Additionally, neural synchrony between the hippocampus and medial prefrontal cortex is essential in memory encoding and retrieval. For example, hippocampal and prefrontal theta become coherent during problem-solving tasks, including those that involve spatial navigation, associative learning, and working memory (Jones and Wilson, 2005; Brincat and Miller, 2015; Tamura et al., 2017; Padilla-Coreano et al., 2019).

Finally, recent work has indicated that inter-brain synchrony increases during cooperative problem-solving tasks, as well as during teaching-learning interactions (Dikker et al., 2017; Xue et al., 2018; Bevilacqua et al., 2019; Lu et al., 2019; Reinero et al., 2020), which has implications for both the problem-solving interactions that take place during the choreographic process and the learning experiences that happen in dance training.

### Social

Humans are inherently social creatures, with successful social interactions relying on social-cognitive skills that contribute to our survival and future reproduction (Herrmann et al., 2007). The field of social neuroscience has indicated that successful social interactions require a correct mental representation of the thoughts and feelings of others, which have been studied using Theory of Mind, empathy, or action-observation tasks and have been linked to extant brain regions including the Default Mode Network, Dorsal, and Ventral Attention Networks, and the Frontoparietal Network (Schurz et al., 2020). Dance incorporates many social aspects, with dance training occurring in groups and dance often involving partnering between two or more people. Historically, dance has been viewed as a social behavior. For example, in the 19th century, Charles Darwin hypothesized that dance and music evolved for the purposes of courtship and mating. Later in the 1920s, anthropologists proposed that dance is a form of social order that increases social cohesion and communication and signals group cohesiveness (Christensen et al., 2017; Zaidel, 2018). In a recent study, empathic ability and resting-state functional connectivity of the insula were assessed in dancers vs. healthy controls. The insula is a region of the cerebral cortex located within the lateral sulcus that is involved in sensorimotor integration (i.e., the transfer of sensory information into motor action) and the generation of subjective emotional states (Craig, 2009; Menon and Uddin, 2010). This region supports feelings of empathy, including empathy for pain, anxiety, social exclusion, disgust, and taste (Jabbi et al., 2007, 2008; Prehn-Kristensen et al., 2009; Mazzola et al., 2010; Masten et al., 2011). Compared to controls, dancers displayed higher levels of empathic ability and higher levels of functional connectivity between the insula and other regions, including the anterior cingulate cortex, middle cingulate cortex, middle temporal gyrus, and medial frontal cortex (Gujing et al., 2019). Also, positive associations were seen between the level of empathy and the level of functional connectivity between the posterior insula and middle cingulate cortex, indicating that these areas may be integral in supporting socioemotional abilities in dancers (Gujing et al., 2019). Mirroring practices in dance and Dance Movement Therapy (DMT) are thought to drive the enhanced empathic abilities in dancers, with this ability being linked to increased activation in the mirror neuron system (McGarry and Russo, 2011).

Social neuroscience has attempted to identify the neural mechanisms underlying social interactions using the hyperscanning technique (Montague et al., 2002). A recent review suggests that social interactions are goal-directed, requiring an increased attunement and allocation of attention to the attainment of “successful and lucrative social interactions,” which are driven by inter-brain synchrony between both the temporoparietal junctions and prefrontal cortices (Gvirts and Perlmutter, 2020). Hyperscanning studies investigating shared intentionality in social interactions have been studied in several real-world tasks. For example, interpersonal brain synchrony increases during such tasks as cooperative game playing, coordinated walking, group humming, guitar playing, and problem-solving (Lindenberger et al., 2009; Cui et al., 2012; Jiang et al., 2012; Müller et al., 2013; Osaka et al., 2014; Nozawa et al., 2016; Ikeda et al., 2017; Pan et al., 2017; Liu et al., 2017; Xue et al., 2018; Lu et al., 2019). One study utilized hyperscanning EEG recordings during natural social interactions between male-female pairs (Kinreich et al., 2017). Compared to a rest condition, during social interactions, neural synchrony [defined here as the correlation of the EEG power spectrum] in the gamma band (30–60 Hz) increased in temporal-parietal brain regions, with neural synchrony being significantly higher in romantic couples compared to strangers. Interestingly, these neural synchrony patterns were significantly associated with periods of social gaze, the experience of positive affect, and the sense of attachment that was experienced with the partner. Considering that gaze and affective expression mark the first emergent nonverbal social behaviors between infants and parents, the authors suggest that brain-to-brain synchrony may underlie social connectedness and attachment (Kinreich et al., 2017). In fact, enhanced brain synchrony has been seen in parent-child interactions, with greater synchrony corresponding to enhanced emotional connectivity between the parent-child pair (Reindl et al., 2018). Brain-to-brain synchrony has also been seen in the classroom, both between students and between teacher and student, with enhanced brain synchrony relating to both class engagement and improved social dynamics (Dikker et al., 2017; Bevilacqua et al., 2019). Finally, an exciting new fMRI hyperscanning study in expert dancers found that various brain regions in social networks were activated during leading, following, mutual partnering, and improvisation conditions; however, neural synchrony was not explicitly examined (Chauvigné and Brown, 2018; Chauvigné et al., 2018). Future hyperscanning studies are warranted to examine how neural synchrony changes relate to the social interactions between dancing individuals, both during a choreographic and improvisational dance. We hypothesize that neural synchrony changes may differ between dance forms with a focus on intentional synchrony (i.e., choreographed dance) vs. spontaneous or emergent synchrony (i.e., improvisational dance; Sgorbati, 2012; Rennung and Göritz, 2016; von Zimmermann et al., 2018).

### Emotional

Proper emotional development throughout childhood and adolescence is critical for healthy emotional regulation and mental states to emerge in adulthood, with these behaviors depending upon correct connectivity of frontoamygdala circuitry (Casey et al., 2019). As a performance art form, dance has an intimate connection to emotion. Dance can be viewed as a form of emotional self-expression through bodily gestures and movements (Arnold, 1986; Ritter and Low, 1996; Winters, 2008). Additionally, dance training often involves emotional interactions with others, such as in the case of working with a dance partner or troupe. Dance performance can also elicit intense emotional reactions for dancers and audience members alike. Using Laban Movement Analysis, researchers have shown that specific movement patterns (in the absence of facial expressions) elicit similar emotions (e.g., happiness, sadness, fear, or anger) for both the dancer and observer (Shafir et al., 2013, 2015; Melzer et al., 2019). The emotional aspect of dance is thought to be the crucial element regarding the beneficial effects of DMT, and dancers show heightened emotional intelligence over non-dance counterparts (Jeong et al., 2005; Punkanen et al., 2017; San-Juan-Ferrer and Hípola, 2020).

Emotional expression and perception, which often rely on nonverbal cues such as facial expression, body movement, and vocal tone, enable us to both share our internal affective state and understand that of another (Symons et al., 2016). This sharing of emotions or emotional contagion enables us to understand the thoughts, intentions, and actions of others and supports interpersonal coordination. At the individual level, emotional detection, integration, and evaluation have been linked to enhanced theta and gamma synchronization in brain regions, including the orbitofrontal cortex, superior temporal sulcus, and amygdala (Symons et al., 2016). Research has also shown that when two individuals are exposed to similar types of emotional experiences, such as emotional movies, neural synchrony between those individuals increases in sensory cortical areas and limbic regions (amygdala, insula, and thalamus), with the intensity of the emotional experience being correlated to the level of neural synchrony (Hasson et al., 2004; Nummenmaa et al., 2012; Kinreich et al., 2017). Of relevance, when viewing an emotionally evocative dance performance, dancers demonstrate greater theta phase synchrony at fronto-central brain regions compared to non-dancers (Poikonen et al., 2018).

### Rhythmic

Early in development, parents communicate with their infants through rhythmic interactions such as rocking, bouncing, patting, singing, and motherese/parentese. These rhythmic communications are thought to enhance interpersonal coordination and neural synchrony between parent and child (Markova et al., 2019) and are critical for the proper development of auditory processing, language, and communication skills (Fujii and Wan, 2014). In fact, abnormalities in neuronal oscillatory activities are prominent in disorders of language pathology, such as autism and dyslexia (Gandal et al., 2010; Goswami, 2011; Heim et al., 2011; Edgar et al., 2015; Murphy and Benítez-Burraco, 2017), and rhythmic skills training has been implemented as a clinical tool to help children with such deficits (Miendlarzewska and Trost, 2013). Dancing involves rhythmicity or the ability to synchronously entrain the body’s movements to musical rhythms. This rhythmic ability emerges early in life, with infants showing the ability to move in sync to musical rhythms (Phillips-Silver et al., 2010). Through dance training, these abilities are enhanced, with dancers being able to entrain more effectively than non-dancers to both musical rhythms as well as the body movements of other individuals (Washburn et al., 2014; Miura et al., 2015, 2016; Jin et al., 2019).

Early studies demonstrated that the brain can become entrained to both visual and auditory rhythmic stimuli, termed photic and auditory driving, respectively (Neher, 1961, 1962; von Gizycki et al., 1998). For example, drumming at a tempo of ~7–9 beats per second can induce theta activity in the brain, which reportedly induces intense, hallucinogenic-like psychological states (Walter and Walter, 1949). Commercial endeavors have utilized this idea to create soundscapes intended to entrain the brain to particular rhythms to induce various psychological states (e.g., relaxation; problem-solving; insight). Additionally, autohypnotic states (driven by rhythmic movements) have also been observed in traditional ritual dances across cultures. For example, trance and possession states have been documented in Haitian Vodou dances, as well as in the traditional Javanese dances of Bali. One study revealed that whole brain alpha activity and frontal midline theta activity increased during a professional dancer’s recall of Salpuri, a shamanic Korean dance form intended to wash away evil spirits (Park et al., 2002). Neuroimaging studies also show that rhythmic music, compared to a scrambled control, generates both intra- and inter-brain synchrony in various brain regions, including subcortical and cortical auditory areas, frontal-parietal attention networks, and motor planning areas (Abrams et al., 2013; Farbood et al., 2015; Kaneshiro et al., 2020). Group music playing has also been shown to induce physiological synchrony (heart interbeat interval) between members of the group, with this effect correlating to perceived group cohesion (Ilanit et al., 2020). One study found that joint guitar playing of a set melodic phrase induced intra- and inter-brain synchrony, as measured by the Phase Locking Index and Interbrain Phase Coherence, respectively, especially at fronto-central electrode sites and most prominently in the theta frequency band (3–7 Hz; Lindenberger et al., 2009). Excitingly, the finding of enhanced intra- and inter-brain synchrony was extended to improvisational guitar playing (Müller et al., 2013), which has implications for improvisational dance forms.

### Creative

Humans are a highly creative species; we value novelty and place a high value on novel ideas. From an evolutionary perspective, creativity is important because it helps drive human progress and can be evidenced throughout society in diverse pursuits including art and science (Wiggins et al., 2015). Creativity is a skill that emerges throughout development, and recent educational efforts have focused on cultivating creativity as an important skill in the classroom and beyond (Kupers et al., 2019). Dance has a major focus on creativity, as the creative process is what makes dance an art form rather than just a standard physical activity. Research has demonstrated that dance enhances creative abilities, with creativity emerging in dance forms associated with high levels of self-expression, such as contemporary dance and improvisation (Arnold, 1986; Fink et al., 2009; Fink and Woschnjak, 2011).

Creativity has traditionally been studied using tests of either convergent or divergent thinking, with engagement in these tasks being associated with activation of the default mode network, including the medial prefrontal cortex and posterior cingulate cortex (Mayseless et al., 2015), as well as increased alpha power in bilateral frontal and right posterior cortical sites (Fink and Benedek, 2014). Newer research demonstrates a U-shaped function during creative thinking whereby alpha power significantly increases at the onset and crystallization of the creative thought (Rominger et al., 2019). Additionally, the moment of intuition or the “AHA moment” has been associated with an increase in gamma synchronization in the right anterior superior temporal gyrus (Jung-Beeman et al., 2004). One study, comparing novice to professional dancers, examined brain activity during both a standard test of creativity and the vivid imagining of improvised dance (Fink et al., 2009). During both creative thinking tests, professional dancers demonstrated significantly increased alpha synchronization compared to novice dancers, especially in posterior parietal areas (e.g., centroparietal, parietotemporal, and parietooccipital electrode sites; Fink et al., 2009). Additionally, recent studies using fNIRS hyperscanning found that interpersonal brain synchronization increased in both the right dorsolateral prefrontal cortex and the right temporal-parietal junction during a cooperative divergent thinking task (Xue et al., 2018; Lu et al., 2019). These findings have implications for group dance improvisation, suggesting that collaborative creativity tasks (such as that which occurs in improvisation) increase neural synchrony between the individuals sharing in the intentional generation of creative ideas.

### Dance Enhances Intra-brain Synchrony

In line with The Synchronicity Hypothesis of Dance and taking into consideration the above findings, certain evidence indicates that dance enhances intra-brain synchrony. First, dancers compared to musicians or laymen show increased theta phase synchrony over fronto-central electrodes (FC3 to FCz; FC3 to FC4; FC5 to FCz; and FC5 to FC4) while watching a dance video (Poikonen et al., 2018). The authors suggest that this increased synchrony may reflect cognitive and/or affective skills that develop as a result of dance training (Poikonen et al., 2018). Second, professional, compared to novice dancers show greater alpha synchronization both during the Alternate Uses Task, a task of creativity, and during imagining of an improvisational dance (Fink et al., 2009). Third, during resting brain states, dancers show heightened power in higher brain frequencies (alpha and beta) compared to professional fastball sports athletes and healthy controls (Ermutlu et al., 2015). Fourth, interventional studies using EEG have shown that compared to a cognitive training control, dance training (24 weeks of a 60-min traditional Greek dance program, twice per week) enhances optimal network performance as assessed by the small-world property, indicating faster information flow and more accurate information integration between distant cortical sites (Zilidou et al., 2018). Considering that these changes showed a positive correlation with improvements in body flexibility suggests that more efficient functioning of neural networks may underlie dance-related physical improvements (Zilidou et al., 2018).

## The Effects of Dance on Brain Structure and Activity

**Figure 5:**
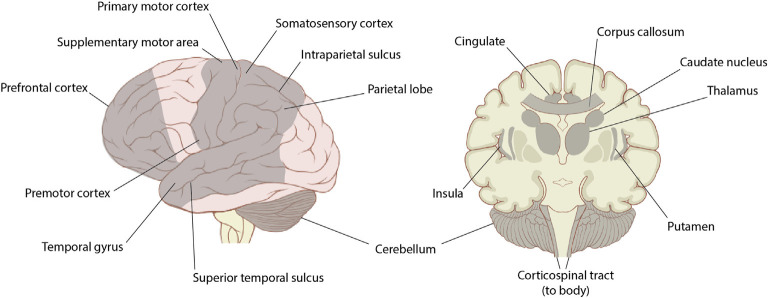
Lateral view and the coronal section of the brain with regions shown to be affected by dance highlighted in gray. Previous literature has reported that dance affects the prefrontal cortex (including ventral medial PFC, medial PFC, and dorsolateral PFC), motor areas (including the primary motor cortex, supplementary motor area, and premotor cortex), somatosensory cortex, temporal areas (including superior temporal gyrus, middle temporal gyrus, and superior temporal sulcus), parietal areas (including superior parietal lobe, inferior parietal lobe, and intraparietal sulcus), cerebellum (including anterior cerebellar vermis and lateral cerebellum), cingulate (including ventral anterior cingulate cortex, ventral medial cingulate cortex, posterior cingulate, and cingulate motor area), caudate nucleus, thalamus, insula, putamen, corpus callosum, and the corticospinal tract. Image source by Patrick J. Lynch *via* Creative Commons.

The research surrounding the effects of dance on brain function, structure, and activation has primarily focused on comparisons between professional dancers and non-dancers, with the majority of these studies being cross-sectional in nature. Due to current technological limitations that hinder the ability to study the moving brain, previous research has examined the brain structure of dancers using MRI, examined brain function while viewing dance or performing minimal movements of the feet or hands using fMRI, or imaged the brain during simple dance video games using fNIRS. This section explains the brain regions most consistently activated or influenced by dance (Figure 5), which will help elucidate potential regions of interest for future studies investigating the effects of dance on intra- and/or inter-brain synchrony.

### Brain Structure in Dancers

Studies have sought to investigate the structural brain changes that result from dance, primarily using comparisons between expert vs. novice or non-dancers. A recent study comparing contemporary dancers (with a mean of 15.3 ± 5.2 SD years of training in styles including ballet, tap, jazz, swing, and ballroom dance) to non-dancers found that dancers have a greater cortical thickness in superior temporal regions (Karpati et al., 2017). Furthermore, gray matter thickness was positively associated with accuracy on tasks of dance imitation, rhythm synchronization, and melody discrimination (Karpati et al., 2017). Greater gray matter volume in the foot regions of the primary somatosensory and motor cortices has also been seen in ballet dancers (Meier et al., 2016).

In a comprehensive study, Burzynska et al. (2017) examined the brains of expert dancers using diffusion tensor, morphometric, resting state, and task-related fMRI, along with a broad neuropsychological task battery and objective measurements of dance skill. They examined 20 dancers with an average of 12 years (± 6 SD) of various dance training and currently dancing 14 h per week (± 8 SD) and compared them to non-dancers, matched for age, BMI (all normal weight), and education. Compared to non-dancers, dancers performed significantly better on measures of balance (measured *via* time spent balancing on one leg) and dance ability (measured *via* a percentage of correct movements), with years of dance experience showing a positive relationship to motor ability. Surprisingly, no between-group differences were seen across a variety of cognitive domains, including fluid intelligence, processing speed, spatial working memory accuracy, working memory span, and task switching. Additionally, no between-group differences were seen in cortical thickness or in subcortical gray or white matter volumes. Interestingly, dancers showed decreased fractional anisotropy (FA) measures in the corticospinal tract, which is a white matter tract that controls the movements of the trunk and limbs—areas important for whole-body movements that are essential in dance. FA approximates the integrity and organization of the brain’s white matter, and others have shown similar findings in dancers in regions such as the corpus callosum and sensorimotor pathways (Hänggi et al., 2010). This finding has also been shown in other highly motor-trained individuals, such as world-class gymnasts (Huang et al., 2015). An additional study comparing expert dancers, expert musicians, and non-dancer/non-musician controls found that dancers had significantly reduced anisotropy (measured via FA and mode of anisotropy) and increased diffusivity (measured via radial diffusivity, axial diffusivity, and mean diffusivity) in the corticospinal tract, superior longitudinal fasciculus, and corpus callosum. These findings are most likely because large axon diameter is associated with increased radial diffusivity (Barazany et al., 2009), which can lead to reduced FA (Fieremans et al., 2008), possibly because large axons are less densely packed than small axons, leaving larger regions of extracellular space surrounding the myelin sheath (Beaulieu, 2002; Barazany et al., 2009; Alexander et al., 2010). Similarly, resting-state studies have shown that compared to non-dancers, dancers demonstrate increased functional connectivity between the middle cingulate cortex and bilateral putamen and between the precentral and postcentral gyri, with these effects being positively associated to the amount of dance training, indicating that dance enhances communication in cortical-basal ganglia loops that govern motor control (Li et al., 2015a).

Collectively, this work indicates that dance training leads to structural brain changes primarily in sensory and motor regions as well as connections between these regions, providing faster conduction and enhanced coordination between these brain areas critical for the expression of dance.

### Action Observation in Dance

Through technique practice, dancers are trained to understand where the body is located in space, navigate and coordinate actions through space, carefully assess the actions of another mover, and engage in a full spectrum of physical movements, ranging from gross motor to fine-tuned actions. Dancers need to develop the ability to quickly and effectively learn choreographic sequences from another dancer, often a teacher or fellow dancer, and execute these prescribed movements with ease and accuracy. Recent research indicates that the action observation network or mirror neuron system may be integral to this ability as well as the ability to perceive others’ emotional landscape (McGarry and Russo, 2011). As many current human neurophysiological and neuroimaging techniques like EEG and fMRI are very sensitive to motion artifacts, recordings are often obtained when the individual is sitting or lying still. Therefore, recording brain activity while dancing has proven difficult; however, researchers have overcome this hurdle by recording the brain activity of dancers while imagining themselves dancing or watching other dancers perform.

The action observation network supports the observations and simulation of others’ movements and behaviors. This network involves brain regions such as the premotor and parietal cortices, as well as the supplementary motor area, superior temporal sulcus, and primary motor cortex. A collection of work has examined the neural correlates of how embodied movement is represented in the brain (Calvo-Merino et al., 2005; Cross et al., 2006). Some of the first fMRI studies in dancers revealed increased activity of the action observation network when viewing more familiar movements (Gardner et al., 2015). A study of trained ballet and capoeira dancers (Calvo-Merino et al., 2005) found greater bilateral activation in motor regions of the action observation network, including the premotor cortex, intraparietal sulcus, right superior parietal lobe, and left posterior superior temporal sulcus when the dancers viewed their own style of dance (e.g., ballet or capoeira) compared to other styles. However, no such familiarity effect was seen in areas related to visual familiarities, such as the fusiform gyrus (Calvo-Merino et al., 2005). Also, a study found that expert ballroom dancers displayed greater activation in the ventral premotor cortex while viewing ballroom dance videos than novice ballroom dancers (Pilgramm et al., 2010). Together, this work indicates that motor familiarity or expertise moderates the activation of the action observation network, specifically, the regions associated with motor function.

This cross-sectional work paved the way for additional interventional studies. In one of the first longitudinal studies using dancers, the brains of 10 expert dancers (mean history of dance training: 12.8 ± 5.6 years) were observed weekly using fMRI as they watched another dancer and imagined themselves performing complex movement sequences that they were currently learning through a rehearsal process (Cross et al., 2006). Specifically, dancers were instructed to imagine themselves performing the dance sequences they were watching and assess how well they could perform these movement sequences. During the 5 weeks of the experiment, the time spent learning these choreographic sequences was 5.2 ± 0.9 h per week. When the dancers observed others performing these choreographic sequences, brain regions of the action observation network, including the inferior parietal lobe, cingulate and supplementary motor areas, ventral premotor cortex, superior temporal sulcus, and primary motor cortex, became active. Also, when compared to watching non-rehearsed, control movements, watching rehearsed movements elicited more pronounced activity in the superior temporal sulcus, ventral premotor cortex, intraparietal sulcus, and supplementary motor area, all within the left hemisphere. Finally, several additional areas emerged—the intraparietal sulcus, inferior parietal lobe, ventral premotor area, and parahippocampal gyrus—that related to the perceived ability of the dancers to perform the actions. Similar findings were also seen in non-dancers with only 5 days of training on a dance video game (Cross et al., 2009). These results suggest that as movements become embodied (that is, as we understand how to seamlessly execute complex movement patterns), the brain develops patterns of activity that represent these learned movements.

Together, these studies indicate that dance enhances the action observation network or mirror neuron system, which may be a key link between sensorimotor training and social cognition. It is through this network that we can process and interpret other’s actions and emotions (Cross et al., 2009; Caspers et al., 2010; Gardner et al., 2015). When we watch others, we glean information about their goals and intentions, which enables us to predict their future behaviors (Blakemore and Frith, 2005; Falck-Ytter et al., 2006; de C Hamilton and Grafton, 2006; de C Hamilton, 2013). Therefore, from an evolutionary perspective, dance may serve to enhance brain networks that support our ability to understand others, i.e., our interpersonal coordination skills.

### The Body and Brain in Motion

Technical limitations make studying the body and brain in motion a difficult task; however, several researchers have taken on this challenge using either limited physical movements (e.g., footwork) or brain imaging devices less prone to movement artifacts (e.g., fNIRS). These studies have revealed that different aspects of dance, such as leading, following, coordinating movements, synchronizing movements to a rhythm, and improvisation, are supported by distinct brain regions that underlie sensory, motor, cognitive, and motivational abilities.

Using positron emission tomography, researchers were able to image the brain while amateur dancers performed tango steps on an inclined surface (Brown et al., 2006). They examined three aspects of dance: entrainment, meter, and patterned movement. The anterior cerebellar vermis, involved in the coordination and accuracy of movement, supported the dancers’ ability to entrain their movements to a musical tempo (compared to moving at a self-paced tempo without music). Dancing to a regular, metric rhythm (compared to moving to an irregular rhythm) was supported by the right putamen, a region of the basal ganglia involved in voluntary movement. Finally, dancing compared to a simple rhythmic contraction of the leg muscles led to greater activation in the medial superior parietal lobe, an area involved in spatial orientation.

Another group took the approach of using fNIRS while non-experienced dancers played a dance video game (similar to Dance Dance Revolution^TM^; Tachibana et al., 2011). They found that compared to rest, dance activates the superior temporal gyrus and the superior parietal lobe, which are two areas involved in sensory-motor integration. Further, the activation in these areas increased as the dance steps became more challenging. A similar study found that temporal accuracy of the dance steps positively correlated with activity in the medial temporal gyrus and suppression of the frontal-parietal cortex (Ono et al., 2014). This correlation suggests that as dancers become more skilled in responding to musical rhythms, they may be more guided by bottom-up rather than top-down cortical activation (Ono et al., 2014). These neural processes may contribute to a flow state (Nakamura and Csikszentmihalyi, 2014) or ease of executing steps as one develops expertise in dance and musicality.

An exciting new line of research has focused on the duetting brain. The brains of expert couples dancers (trained in Argentine tango, salsa, swing, or ballroom) were imaged with fMRI while another dancer engaged in bimanual movements of the wrist and metacarpophalangeal joints in all three planes of motions, with contact occurring at the inner surfaces of the fingers (Chauvigné et al., 2018). Interestingly, the brain showed distinct activation patterns during different periods of dancing, namely, leading, following, coordinating movements together in a pre-learned sequence (a mutual condition), and improvising. Leading was orchestrated by brain regions involved in spatial orientation, sensorimotor integration, motor planning and sequencing, the initiation of motor movements, and error correction, including the primary motor cortex, premotor cortex, cingulate motor area, supplementary motor area, superior parietal lobule, inferior frontal gyrus, lateral cerebellum, and superior temporal gyrus (Chauvigné et al., 2018). Following was orchestrated by more sensory-focused brain regions, including those that regulate tactile perception and proprioception, motion tracking, social cognition, and the monitoring of outcomes concerning reward, including the sensory-motor cortex, sensory thalamus, MT+/V5 (motion area of the middle temporal region), posterior superior temporal sulcus, ventral anterior cingulate cortex, ventral medial prefrontal cortex, caudate nucleus, and nucleus accumbens (Chauvigné et al., 2018). Coordinating movements together in a pre-learned sequence (i.e., the mutual condition) was regulated by higher cognitive brain regions, including the medial prefrontal cortex, posterior cingulate cortex, and temporoparietal junction, which suggests that this type of interaction in dance requires a high degree of “awareness of the thoughts and intentions of the partner” (Chauvigné et al., 2018). Finally, improvisation (defined as the *de novo* generation of non-learned sequences) activated a similar set of brain regions as the leading condition but activated the additional areas of the dorsolateral prefrontal cortex and bilateral putamen—areas involved in decision-making and motivation.

Collectively, these neuroimaging studies suggest that dance training may lead to the reorganization of brain systems that support expert dance skills. Figure 5 summarizes the brain regions affected by dance, which include motor, somatosensory, and emotional processing regions, as well as the action observation network. The brain changes seen in dancers may be a result of years of training in the development of the proprioceptive, motor movement, and coordination systems. Future studies are needed to investigate the effects of dance training on the volumetric, oscillatory, and functional changes associated with other brain regions, including the prefrontal cortex, hippocampus, basal ganglia, amygdala, and cerebellum—regions associated with executive functioning, memory, movement and motivation, emotion, and coordination, respectively.

## Discussion: Conclusions, Clinical Utility, and Future Directions

Dance is a complex physical activity that engages a dynamic network of brain regions, including regions that support sensory, motor, cognitive, social, emotional, rhythmic, and creative behaviors. In this Hypothesis and Theory article, we cultivated a definition of dance that encompasses diverse movement practices that are exhibited across various cultures and that are engaged in for the purposes of ritual, performance, or social interactions. Further, we identified and demonstrated six universal movement patterns, based on the Bartenieff Fundamentals, that are directly linked to neurodevelopment: breath, naval radiation (core-distal), spinal connection (head-tail), homologous connection (upper-lower), homolateral connection (body half), and contralateral connection (diagonal). We further discussed how the benefits of dance to the brain are multifaceted, with the most prominent finding being that dance leads to larger axonal diameters, especially in the white matter tracts connecting sensory to motor regions and connecting one brain hemisphere to the other. These enlarged axonal diameters suggest that dance may help support a faster speed of conductivity, which could enhance the brain’s ability to communicate information between different brain regions or networks. Critically, we offer a testable hypothesis (Figure 2) that suggests that through dance, neuronal populations of the brain become highly synchronized, which supports rhythmic coordination between different brain regions (e.g., those that support sensory, motor, cognitive, or emotional abilities) and thus more fluid or effective communication between these networks. We suggest that humans may engage in dance to generate these brain states, which cultivate a pleasurable or positive affective state, and which in turn may motivate us to engage in the behavior. Further, we speculate that dancing together cultivates not only intra-brain synchrony but also inter-brain synchrony, which can occur between two individuals (e.g., in dancing with a romantic partner) or as part of a group (e.g., in ritualistic dance or a dance troupe) and serves to drive interpersonal coordination.

### Clinical Utility

From a clinical perspective, the current hypothesis suggests that dance may be especially helpful for treating disorders with impairments in brain oscillations (Başar and Güntekin, 2008; Başar, 2013). Autism spectrum disorder (ASD) is an example of one such disorder. ASD is characterized by difficulties with voluntary motor activity, social interaction, and sensory modulation, and disturbances of early developmental movement patterns (e.g., the inability to effectively cross the midline of the body or deficits in contralateral movements) have been shown to predict ASD development later in life (Teitelbaum et al., 1998; Harris, 2017; Sacrey et al., 2018). These behavioral difficulties are thought to arise from abnormal cortical oscillations during development as well as deficits in the mirror neuron system (Dapretto et al., 2006; Bernier et al., 2007; An et al., 2018; Buard et al., 2018; Hinkley et al., 2019). Specifically, individuals with ASD have abnormal activity in the motor-related alpha mu, beta, and gamma signals. Mirroring, physical synchronization, rhythm, and social reciprocity are inherent behaviors in dance (DeJesus et al., 2020), and Ramachandran and Seckel (2011) have suggested that a dance intervention with an emphasis on mimicry may activate the “dormant” mirror neuron system in individuals with ASD. We hypothesize that reengaging developmental movement patterns in dance may be helpful to repattern oscillatory activity, leading to clinical improvements in ASD and other disorders with oscillatory activity impairments.

To date, several studies have explored the therapeutic effects of dance and DMT in clinical populations, most notably in Parkinson’s disease (Hackney et al., 2007; Sharp and Hewitt, 2014; McNeely et al., 2015). In addition to movement disorders, dance/DMT is effective in improving symptoms of cognitive, social, and emotional disorders, such as dementia (Hokkanen et al., 2008), ASD (DeJesus et al., 2020), and depression (Karkou et al., 2019). This work suggests that dance may be an effective complementary treatment for a variety of clinical conditions, but further study is needed to replicate these findings in larger and more well-controlled trials. Future studies are also needed to elucidate the neural mechanisms driving dance-induced behavioral improvements, the most effective components of dance interventions, and the optimal interventional designs for clinical application.

### Future Directions

What does the dancing brain look like? The research to date has paved the way to understand the interacting, dancing brain and how the brain supports coordinated interactions (both physical and emotional) between bodies in space. Future work is warranted to establish whether coordinated actions in the body (and between bodies) are evidenced as coordinated rhythms in the brain and how these rhythms of body and brain support brain functioning. The exploration of how dance influences intra- and inter-brain synchrony will require simultaneous recordings of the body and brain in motion (or bodies and brains if exploring inter-brain synchrony). Inter-brain dynamics could be explored between two or more dancers, as occurs in partner dancing or a troupe of dancers. Brain activity should be captured through techniques such as EEG or fNIRS, while body movement should be captured through motion capture systems, ideally, both being wireless. These body-brain recordings will need to be temporally paired on a millisecond timescale such that the relationship between these measures can be examined. Additional studies could focus on the inclusion of musical rhythms as an additional parameter to examine.

Technological limitations in neuroscience make studying intra- and inter-brain synchrony in dancing individuals a challenging area to investigate, but collaborations between electrical engineers and neuroscientists have set the stage for the development of wireless, wearable technologies (Lin et al., 2010; Liu et al., 2018b; Radüntz and Meffert, 2019). Advancements in the field of mobile brain/body imaging (MOBI; Cheron et al., 2016; Jungnickel and Gramann, 2016; Brantley et al., 2018; Gennaro and de Bruin, 2018; Jungnickel et al., 2019) will be necessary to explore the current hypotheses. Future studies optimizing technological strategies for imaging the moving brain will be needed to identify how the brain supports individual and group dance and movement practices. Several technological steps must be taken. The first step is to develop technologies to enhance the signal-to-noise ratio during movement. fNIRS may be a promising technique for this purpose, as it is portable, inexpensive, and less sensitive to motion artifacts than fMRI (Scarapicchia et al., 2017). fNIRS has been successfully used to record brain activity during freely-moving activities, such as walking and bicycle-riding (Piper et al., 2014; McKendrick et al., 2017). Additionally, filtering methods may be used to further minimize the noise from motion artifacts (Izzetoglu et al., 2010; Robertson et al., 2010). The second step will be to coordinate measurements between body movements and brain changes. Yet a third step will be to coordinate body and brain measurements between two or more dancers. Finally, a fourth step will be to expand this work by taking it out of the laboratory and into a real-world performance space.

### Final Remarks

Here we cite evidence that the neurobehavioral functions involved in dance or dance itself enhance both intra- and inter-brain synchrony. Through dance, we can drive neural synchrony at both the individual and group levels, which causes increased interpersonal coordination skills and enables us to feel more connected to the individuals in our social circles. This is a direct example of we-intention or shared intentionality (Hasan and Kayle, 2020) and has direct implications for how we view our conscious experience. Consciousness can be redefined not just as an individual process but a shared experience and we as individuals can influence the consciousness of others through our shared experiences (Valencia and Froese, 2020). Dance is one way to enhance the collective conscious experience and drive it towards a shared, pleasurable reality.

## Data Availability Statement

The original contributions presented in the study are included in the article, further inquiries can be directed to the corresponding author.

## Author Contributions

JB conceived the work, along with the neurocentric definition and hypothesis of dance; developed the progression of the article; wrote the abstract, introduction, and discussion, along with other main sections; and edited the manuscript in full. MS developed and edited sections throughout the manuscript. RR developed and wrote sections pertinent to the evolutionary and cultural aspects of dance. All authors contributed to the article and approved the submitted version.

## Conflict of Interest

The authors declare that the research was conducted in the absence of any commercial or financial relationships that could be construed as a potential conflict of interest.
